# Behavior of “Intermediate” Males of the Dimorphic Squid *Doryteuthis pleii* Supports an Ontogenetic Expression of Alternative Phenotypes

**DOI:** 10.3389/fphys.2019.01180

**Published:** 2019-09-13

**Authors:** Lígia H. Apostólico, José E. A. R. Marian

**Affiliations:** Department of Zoology, Institute of Biosciences, University of São Paulo, São Paulo, Brazil

**Keywords:** alternative reproductive tactics, intrasexual male dimorphism, male–male competition, ontogeny, reproductive success

## Abstract

The expression of alternative reproductive tactics (ARTs) by different-sized males of loliginid squids has been extensively investigated. In loliginids, alternative phenotypes are characterized by discontinuous differences in behavior, body size, sperm deposition site, and morphology and functioning of ejaculates. Large consort males guard females, display agonistic behaviors toward rival consort males, and mate with females in the male-parallel (MP) position. Small sneaker males avoid fighting contests and instead adopt furtive behaviors to access females guarded by consort males, mating with females in the head-to-head (HH) posture. Recently, the reappraisal of preserved material from the loliginid squid *Doryteuthis pleii* showed that intermediate-sized males (so-called “intermediate” males) had both sneaker- and consort-like ejaculates, leading to the hypothesis of them being a transitional stage between both phenotypes. Here, we describe observations made in captivity showing that intermediate males can display agonistic behaviors toward consort males and mate with females in both mating positions, depending on the male’s current reproductive context, i.e., generally in HH, but switching to MP when the female is laying eggs. Such unusual findings of intermediate males simultaneously displaying behaviors of both sneaker and consort males comprise additional evidence corroborating the ontogenetic hypothesis for phenotypic expression of ARTs in this species. Taken together, our results indicate that (1) instead of competing with large consort males for female access and monopolization, small/young males adopt sneaker tactics to obtain mating opportunities, and (2) as they continue to grow, they gradually modify the morphology of their ejaculates and their mating behavior, going through an “intermediate” stage, before becoming large consort males.

## Introduction

Mating behavior of loliginid squids (Loliginidae, Cephalopoda) is notable not only because they exhibit several complex behaviors, including agonistic contests and mate guarding (Hanlon and Messenger, 2018), but also because, for several species, males within the same population can express alternative reproductive tactics (ARTs) when pursuing the fertilization of females’ ova (Supplementary Figure S1) (e.g., Hanlon, 1996; Hanlon et al., 2002; Iwata et al., 2005). Large (consort) males pair with females, protecting them from the harassment of other consort males. Female guarding occurs before and after mating and also while the female is laying eggs (Hanlon et al., 1997; Hanlon, 1998). It consists of a series of agonistic exhibitions toward rival males, including the expression of stereotyped body patterns (e.g., the exhibition of red stripes laterally along the body, referred as “lateral flames”) (Supplementary Figure S1D) and even the engagement in physical bouts with other consort males (DiMarco and Hanlon, 1997). During mating, they place themselves below the females and mate with them in a position called “male-parallel” (MP), inserting their spermatophores inside the female’s body and implanting them near her oviduct opening (Supplementary Figures S1A,E,F). Small (sneaker) males, however, do not display any agonistic behaviors toward other males. Instead, they make attempts of mating stealthily with females guarded by consort males (Hanlon et al., 2002). During mating, they place themselves in front of the female and mate with her in a position known as “head-to-head” (HH), usually without any resistance from consort males, placing their spermatophores near the seminal receptacle of the female, located close to her mouth region (Supplementary Figures S1A–C) (e.g., Hanlon, 1996; Hanlon et al., 1997).

In addition to contrasting behavioral strategies, different mating positions and spermatophore attachment sites (Supplementary Figure S1), male alternative phenotypes in loliginid squids also have singularities concerning morphological and physiological traits, particularly related to their body size ranges and to attributes of their ejaculates (e.g., Iwata and Sakurai, 2007; Iwata et al., 2015, 2018; Apostólico and Marian, 2017, 2018a). The overall morphology of spermatophores and spermatangia (i.e., everted spermatophores, implanted in the female during mating) of different male morphs is clearly distinct (Supplementary Figures S1C,F,H,I) (e.g., Iwata et al., 2015; Apostólico and Marian, 2017). They may also diverge, for example, in terms of spermatophore size and sperm size, volume, and swimming behavior (e.g., Iwata et al., 2011; Hirohashi and Iwata, 2014; Apostólico and Marian, 2017, 2018a). Sneaker males, for instance, produce smaller spermatophores, with less but longer sperm, which also have the unique ability to swarm after release, a feature not observed so far in any consort sperm (e.g., Iwata and Sakurai, 2007; Hirohashi et al., 2013, 2016; Apostólico and Marian, 2017, 2018a).

Expressing one or the other male phenotype can either represent an immutable pathway, meaning that individuals will always play one or the other mating tactic throughout their entire life, or it can be a reversible pathway, meaning that males may shift from one tactic to another once or even interchange between them multiple times in life (Taborsky et al., 2008). Although limited to a few number of studies, several mechanisms responsible for the expression of ARTs have been proposed for different loliginid species. While studying the mating behavior of *Doryteuthis pealeii* both in captivity and on natural spawning grounds, Hanlon et al. (1997) proposed that male phenotypic expression should correspond to flexible tactics, as males seemed to be able to switch between sneaker and consort behaviors according to the mating context to which they were momentarily exposed to (e.g., male size compared to those of the rival males around). For males of *Heterololigo bleekeri*, on the other hand, Iwata and Sakurai (2007) suggested that the expression of male phenotypes should represent permanent tactics. And finally, Mather (2016) observed that early adult males of *Sepioteuthis sepioidea* adopted sneaker tactics on natural environments, changing to consort tactics later in life as they grow.

Congruently with the last hypothesis, a detailed study on this matter focusing on *Doryteuthis pleii* also advocated the plausibility of an ontogenetic shift between divergent male morphs in the species (Apostólico and Marian, 2018b). The hypothesis was based on the description of males of intermediate size and age within the population, which were then hypothesized as a transitional phase from the sneaker to the consort morph. The so-called “intermediate” males diverged from typical sneaker and consort males due to the presence of sneaker- and consort-like spermatophores – along with peculiar spermatophores that shared intermediate characteristics between both types – in their reproductive system (Supplementary Figures S1H,I; Apostólico and Marian, 2018b).

The distribution of different spermatophore types within the reproductive organ of the same individual suggested the occurrence of a transition from sneaker- to consort-like spermatophores (Apostólico and Marian, 2018b). Therefore, intermediate males of *D. pleii* were considered as a potentially transitional phenotype during a progression from sneaker to consort. However, considering that all information was obtained after revisiting preserved material (Apostólico and Marian, 2018b), any conjecture on their behavior was not possible at that moment. Therefore, in order to continue exploring the unusual aspects of these intermediate males of *D. pleii*, the present study aimed at describing the behavior of these males in captivity, when in the presence of females and rival males. Such information could shed light on the ontogenetic hypothesis for phenotypic expression of ARTs in this species, e.g., if intermediate males indeed comprise a transitional phenotype, we expect they would display a transition from typical sneaker- to typical consort males’ behaviors.

## Materials and Methods

Mature specimens of *D. pleii* were collected along the summer months (January–March) of 2018 and 2019, off São Sebastião Island (between 23°43′56^″^S, 45°17′21^″^W and 23°48′26^″^S, 45°14′27^″^W, São Paulo state, southeastern Brazil). Total experimentation period lasted for 10 weeks (5 weeks in each year). Daily expeditions to the collection site (*N* = 17) lasted about 4 h each day, often from 08:00 to 12:00. At the site, animals were sampled individually by hand-jigging and maintained alive inside the vessel in tanks of 250 L with a continuous flow of fresh seawater. On average, 20 individuals were collected each day, and mortality rate inside the vessel was about 30%. From all collected animals (in both years), a total of 117 females and 124 males survived and were taken to the Center of Marine Biology of University of São Paulo (CEBIMar-USP). There, the animals were placed in tanks of 250–1000 L, with an open seawater system and water temperature from 26 to 29°C, and fed with fresh shrimps *ad libitum*. Males and females were maintained in separate tanks for 24 h before the beginning of the experiments. Only 71 out of 117 females and 66 out of 124 males were maintained for the trials, as the remaining animals either died in the tank or were used for tissue sampling for different approaches in other ongoing studies conducted by the authors.

Prior to the experiments, all males were measured and classified as either sneaker or consort males based on their body size, according to Apostólico and Marian (2018a). Males smaller than 169 mm of mantle length (ML) were assigned as sneaker males, whereas those larger than 169 mm ML were categorized as consort males. Sneaker and consort males were then maintained in different tanks to avoid physical attacks or cannibalism on the smallest individuals. For the trials, sneaker and consort males were assorted randomly from the respective tanks.

Experimental manipulations (*N* = 52) consisted in placing one random female with either (i) one consort male, (ii) one sneaker male, (iii) one sneaker and one consort male, (iv) two consort males, or (v) two sneaker males, and recording body pattern displays and mating positions adopted by each male. Observations were performed everyday, preferably between 07:30 and 18:30 h (i.e., during daylight) and interrupted during the night. Whenever possible, behaviors were recorded using a GoPro HERO + LCD (ca. 1.2 h of recorded behavior). Behavioral terminology followed Hanlon et al. (1994) and DiMarco and Hanlon (1997).

Each female was used only once, but some males were used in more than one trial. In 15 of the 52 conducted trials, there were no relevant interactions between individuals (i.e., neither agonistic behaviors, mating, or spawning) and therefore they were excluded from the results. Experiments lasted from 3 h up to 4 days. They were discontinued either when one of the animals died or when the female spawned. After spawning, females were anesthetized (in a 7.5% solution of MgCl_2_ diluted in seawater) and dissected. The oviduct membranes and the seminal receptacle regions were then inspected under the stereomicroscope for the presence of implanted sneaker-, intermediate-, and/or consort-like spermatangia. Females that died before spawning were not processed and data on which spermatangia types were transferred during mating are lacking.

As intermediate males are characterized by intermediate body size between those of sneaker and consort males, it was not possible to know *a priori* which males were indeed “intermediate males” based solely on ML measurements. As an accurate identification of these individuals requires the analysis of their spermatophores and spermatangia, they were first classified as either “sneaker males” or “consort males” based on their body size for the experiments, with male morph appropriate identification being confirmed later, only after anesthesia and analysis of their ejaculates’ morphology under stereomicroscope (Supplementary Figures S1H,I) (see Apostólico and Marian, 2017, 2018b).

The same method above was also used to confirm male morph identity of sneaker and consort males. Thus, males of small size (ML < 169 mm), which mated only in HH, showed no agonistic behavior, and had only sneaker-like spermatophores/spermatangia (Supplementary Figures S1C,H,I) were confidently assigned (and hereafter called) as sneaker males, whereas those of large size (ML > 169 mm), which adopted only MP mating posture, were aggressive toward rival males, and had only consort-like ejaculates (Supplementary Figures S1F,H,I) were classified (and hereafter called) as consort males. In turn, males of intermediate size (around 169 mm ML), which showed agonistic displays (see the section “Male Agonistic Behavior” in the section “Results”), adopted both MP and HH (see the section “Mating Posture” in the section “Results”), and had different spermatophore/spermatangia morphologies (Supplementary Figures S1H,I) (sneaker-, consort-, intermediate types – see the section “Spermatophore Morphology” in the section “Results”) were labeled (and hereafter called as) intermediate males. Thus, based on body size, mating posture and behavior, and ejaculates’ morphology, our total sample of 66 males was revealed to be composed of 34 consort (178–315 mm ML), 20 sneaker (102–169 mm ML), and 12 intermediate males (132–178 mm ML). The experimental sample (i.e., males selected from the total sample to participate in the trials) was composed of 19 consort males (178–285 mm ML), 6 sneaker males (102–156 mm ML), and 10 intermediate males (132–178 mm ML).

In Brazil, ethics approval is still not required for experimentation with cephalopods by the “Conselho Nacional de Controle de Experimentação Animal” (CONCEA). However, this study has been carried out in accordance with international protocols for the welfare of cephalopods to minimize animal suffering (following Moltschaniwskyj et al., 2007; Butler-Struben et al., 2018).

## Results

Results from the 37 trials are summarized in Table 1. Raw data from each experiment, including information on spermatophore and spermatangia morphology obtained from dissected males (i.e., from their storage organs) and females (i.e., from their oviduct membranes and seminal receptacles), respectively, are presented in Supplementary Tables S1–S3.

**TABLE 1:** Summary of the 37 trials performed with the squid *Doryteuthis pleii* in captivity.

**Trial**	***N***	**Spawning**	**Agonistic behavior**	**Mating posture**
♀ + ♂_*CO*_	4	Yes	–	MP (03–04 h)
♀ + ♂_*SN*_	3	No	–	HH (−)
	3	Yes	–	HH (03–26 h)
♀ + ♂_*IN*_	6	No	–	HH (−)
	1	Yes	–	HH (03–16 h)
♀ + ♂_*SN*_ + ♂_*CO*_	2	No	♂_*SN*_: No (100%), ♂_*CO*_: No (100%)	♂_*SN*_: HH (−), ♂_*CO*_: ?
♀ + ♂_*SN1*_ + ♂_*SN2*_	1	Yes	♂_*SN1*_: No (100%), ♂_*SN2*_: No (100%)	♂_*SN1*_: HH (24 h), ♂_*SN2*_: HH (during spawning)
♀ + ♂_*CO1*_ + ♂_*CO2*_	5	No	♂_*CO1*_: Yes (100%), ♂_*CO2*_: Yes (100%)	♂_*CO1*_: ?, ♂_*CO2*_: ?
♀ + ♂_*IN*_ + ♂_*CO*_	3	No	♂_*IN*_: Yes (67%), ♂_*CO*_: Yes (100%)	♀_*INT*_: HH (−), ♂_*CO*_: ?
	5	Yes	♂_*INT*_: Yes (20%), ♂_*CO*_: Yes (20%)	♂_*IN*_: HH (0h30–48 h) + MP (during spawning), ♂_*CO*_: MP (during spawning-3 h)
♀ + ♂_*IN*_ + ♂_*SN*_	4	Yes	♂_*IN*_: No (100%), ♂_*SN*_: No (100%)	♂_*INT*_: HH (4–32 h) + MP (during spawning), ♂_*SN*_: HH (7–34 h)

*The following criteria were analyzed: (1) “Spawning,” if the female laid eggs after mating (Yes or No). (2) “Agonistic behavior,” if the male showed any aggressive display toward the other male in the tank (Yes or No). Percentage value in parentheses represents the number of trials (from total number) in which agonistic behavior was observed. (3) “Mating posture,” mating position adopted by each male (HH, head-to-head; MP, male-parallel). In parentheses, interval (in hours) between mating and spawning events. See text for a full description of each trial type. Raw data for each experiment are presented in Supplementary Tables S1–S3. ♀, female; ♂_*CO*_, consort male; ♂_*IN*_, intermediate male; ♂_*SN*_, sneaker male; –, not applicable; ?, unknown.*

### Male Agonistic Behavior

**FIGURE 1:**
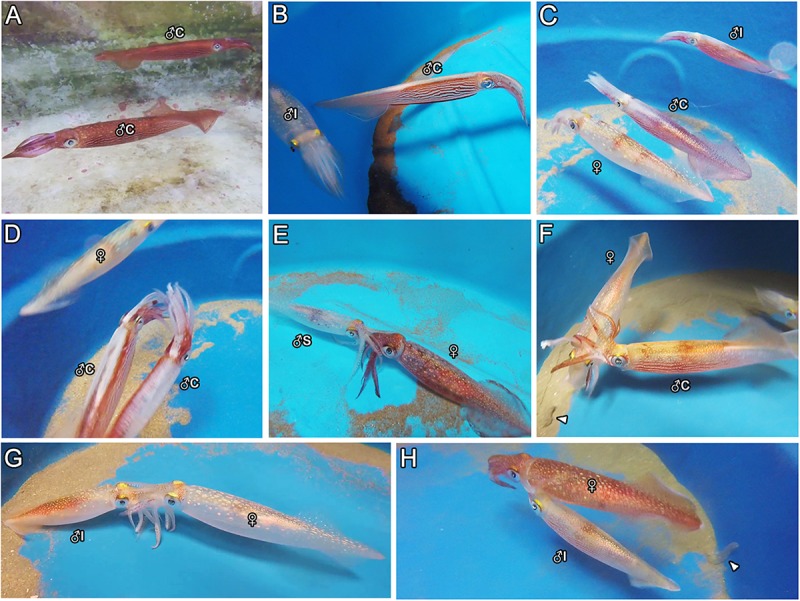
Examples of male agonistic behaviors **(A–D)** and mating positions **(E–H)** observed during the trials. **(A)** Two consort males exhibiting the “all dark” body pattern to each other. **(B)** A consort male displaying “lateral flames” and “mid-ventral ridge” along the body toward a small (intermediate) male. **(C)** An intermediate male trying to fight a large consort male, which is paired with a female. **(D)** Two consort males fighting each other (“fin-beating”). **(E)** A sneaker male mating in head-to-head. **(F)** A consort male mating in male-parallel. **(G)** An intermediate male mating in head-to-head. **(H)** An intermediate male mating in male-parallel. Arrowheads indicate egg strings in panels **(F)** and **(H).** ♀ = female; ♂C = consort; ♂S = sneaker; ♂I, intermediate male.

From all the interactions recorded in captivity, the display of agonistic behaviors between males was certainly the most common during the trials. Aggressive demonstrations advertised by males often started with the exhibition of typical skin colorations, such as the pattern known as “all dark” and the display of “lateral flames” and “mid-ventral ridge” along the body (Supplementary Figure S1D and Figures 1A–C), usually progressing to actual physical disputes, such as “fin-beating” (i.e., males eagerly colliding their fins against each other; Figure 1D). Occasionally, males even tried to attack each other, using their arms in attempts to hold the opponents and hurt them with their beaks. When two males were placed in the same tank, the consort male always displayed such agonistic behavior toward another consort male (Table 1, Figures 1A,D, and trials 14–18 in Supplementary Table S2), and less frequently toward a smaller, intermediate male (Table 1, Figures 1B,C, and trials 26–29 in Supplementary Table S3). On the other hand, none of the sneaker males showed such behaviors toward either smaller or larger males (Table 1 and trials 11–13 and 34–37 in Supplementary Tables S2, S3, respectively). Contrastingly, intermediate males occasionally showed agonistic behaviors, e.g., lateral-flames and fin-beating, toward larger consort males when they were placed together in the same tank (Table 1, Figure 1C, and trials 27–29 in Supplementary Table S3). At that time, these observations were disconcerting, as these males were mostly classified as sneaker males based on their size, but their behaviors were contrasting to those of typical sneaker males.

### Mating Posture

All sneaker males mated only in HH (Table 1 and Figure 1E), despite the mating context, i.e., whether alone with the female or in the presence of another male, and despite female status, i.e., if the female was in the imminence or not of spawning (Table 1 and Supplementary Tables S1–S3). Sneaker males typically swam alone in the tank, i.e., not paired with females, suddenly approaching and grabbing the female with their arms to mate in HH. Also, when the females were present, they were constantly harassing them by mating attempts. Consort males, in turn, usually paired with a chosen female and swam alongside her up to hours and days before mating. They only mated in MP (Table 1, Figure 1F, and Supplementary Tables S1–S3), and mating occurred only close to (i.e., <4 h before) or during spawning (Table 1 and Supplementary Tables S1–S3). So, instead of constantly trying to mate with females before spawning, consort males spend most of their time in protecting the female from other males in the tank.

Similarly to sneaker males, intermediate males did not pair with females, and instead tried constantly to abruptly grasp the female to mate in HH. Differently from sneaker males, though, the mating behavior of intermediate males seemed to depend on their current context (i.e., female status). They frequently performed HH (Table 1, Figure 1G, and Supplementary Table S3), regardless of whether another male was present or absent (either a sneaker or consort male), if spawning by the female has not occurred. However, they were able to switch to consort tactics, i.e., pairing with the female and mating in MP, when the female started laying eggs (Table 1, Figure 1H, and trials 33 and 37 in Supplementary Table S3).

### Female Behavior

Although females were typically passive, they showed hostile behaviors toward sneaker and intermediate males at times along the trials. During constant harassment by these males, females often rejected their mating attempts by displaying the “all dark” body pattern or by escaping through rapid jet-propulsion movements. Rarely, females even tried to bite sneaker and intermediate males swimming around. However, none of these rejection behaviors was shown toward consort males during the experiments.

### Spermatophore Morphology

A conclusive classification of male morph was only possible after dissection of individuals and inspection of their spermatophores and spermatangia (presented in Supplementary Tables S1–S3). Almost all males with typical consort body size (i.e., ML > 169 mm) had only consort-like ejaculates (Supplementary Tables S1–S3), except for the male on trial 19 (Supplementary Table S3), which was revealed to be an intermediate male with a ML of 178 mm. Interestingly though, not all males with typical sneaker body size were in fact sneaker males, being later classified as intermediate males based on their spermatophore morphology (Supplementary Table S3). Among these males, some had only sneaker-like and intermediate spermatophores, whereas others had only intermediate and consort-like ones, or even all three types altogether (Supplementary Table S3).

Also, the peculiar males of intermediate size that had an unusual behavioral combination of agonistic displays (trials 27–29 in Supplementary Table S3) and mating postures (trials 33 and 37 in Supplementary Table S3) were confirmed to be intermediate males. While four of them had only intermediate and consort-like spermatophores, the other one had all three types (sneaker-, consort-like, and intermediate spermatophores) inside its storage organ (Supplementary Table S3).

### Spermatangia Morphology

Inspection of the seminal receptacle and oviduct membranes of females (after spawning) were performed to confirm which spermatangia types (sneaker-like, intermediate, or consort-like; Supplementary Figure S1I) intermediate males transferred during mating in HH and MP, respectively (shown in Supplementary Table S3). Almost all intermediate males that mated only in HH (trials 25, 30–32, and 34–36 in Supplementary Table S3) transferred only sneaker-like spermatophores to the females’ seminal receptacle (Supplementary Table S3). In trial 29, however, an intermediate male mated in HH and transferred intermediate spermatophores to the female (Supplementary Table S3).

Interestingly, intermediate males from trials 33 and 37 transferred only one type of spermatophore, even though they adopted both mating postures (Supplementary Table S3). While in trial 33 the male transferred only intermediate spermatophores on both HH and MP (as intermediate spermatangia were found both in the seminal receptacle and in the oviduct membranes, respectively), the male in trial 37 transferred only sneaker-like spermatophores. In this last trial, however, spermatangia were found exclusively near the seminal receptacle, but not attached to the oviduct membranes. As discussed below, small club-like spermatangia transferred during MP mating could have been already flushed from the female’s mantle cavity by the time females were dissected.

## Discussion

This is the first description of the mating behavior of intermediate males of *D. pleii* in captivity. In this species, so-called “intermediate males” have been previously characterized by intermediate size and age when compared to those of typical sneaker and consort males and by their internal morphology, particularly related to the presence of sneaker-, consort-like, and intermediate ejaculates and sperm with different behavior (i.e., aggregation vs. diffusion) simultaneously within a single individual (Apostólico and Marian, 2018b). Here, it has been shown that these males also manifest a combination of both male morphs’ behaviors in terms of agonistic and mating displays. While typical sneaker males were never aggressive toward other males, independent of their relative size, and typical consort males were always aggressive toward other consort males (and even toward intermediate males sometimes), intermediate males were, in some trials, hostile when in the presence of large consort males. Also, regarding their mating posture, intermediate males preferably mated in HH (ca. 91% of times), resembling sneaker males, but were able to switch to MP, consistent with consort tactics.

Evidence on intermediate males of *D. pleii* simultaneously displaying behaviors of both sneaker and consort males comprise additional evidence supporting that these males may be indeed a transitional stage between both male morphs. According to the ontogenetic hypothesis for phenotypic expression of ARTs in this species (Apostólico and Marian, 2018b), young males adopt furtive tactics as sneaker males while small in size, instead of competing with large consort males for female monopolization. Therefore, they can guarantee mating opportunities and offspring paternity. However, as they continue to grow, they gradually modify the morphology of their ejaculates and their behavior from a sneaker- to a consort phenotype, going through an “intermediate” (morphological and behavioral) stage, before reaching a certain body size, at which they would benefit from adopting dominant tactics as large consort males (see Apostólico and Marian, 2018b). This is also congruent with a previous study on the loliginid squid *S. sepioidea*, in which early adult males play sneaker tactics, but later change to consort tactics as they grow (Mather, 2016).

In the loliginid squid *S. lessoniana*, small males consistently adopt sneaking tactics (Wada et al., 2005; Lin and Chiao, 2018). However, it seems that individuals are able to perform both mating tactics, with male “choice” on the expression of one tactic over the other resulting from visual signals manifested by the female (Lin and Chiao, 2018). When small males try to mate in MP, females consistently reject them through visual body patterns in their skin. However, when these males change to the male-upturned tactic (a sneaking tactic), their success rate is higher, as female rejection is lower (Lin and Chiao, 2018). Here, females of *D. pleii* rejected both sneaker and intermediate males during some of the trials, expressed by quick changes in body color, rapid jet-propulsion movements away from the male, and even aggressive displays. However, no rejection toward consort males was observed. This could be due to behavioral differences between males of different size. While small males (sneaker and intermediate ones) constantly harass the female by recurrently trying to copulate, consort males usually pair with the female but do not engage in mating attempts until the female is close to laying the eggs. However, none of the sneaker males even attempted to mate in MP during the trials, so it is unlikely that they use HH mating because of female rejection signaling in *D. pleii*.

The male behavior of adopting one or another tactic due to female rejection in *S. lessoniana* raises the hypothesis that male squids’ mating strategy is context dependent (Lin and Chiao, 2018). Such male behavioral flexibility has been previously proposed for another loliginid, *D. pealeii*, as small males in this species often play sneaking tactics while in the presence of larger rivals, but switch to MP when the large male is withdrawn from the spawning ground, possibly because fertilization success is higher when adopting MP posture (Hanlon et al., 1997), as explained below. In the present study, however, sneaker and consort males adopted only HH and MP, respectively, and none of them ever attempted to switch between tactics, independently of mating context, i.e., presence or absence of other males (of same or different size). Also, although mating attempts by consort males seem to depend on female status (i.e., in the imminence or not of egg-laying), as they preferably mate with females close to or during spawning, the behavior of sneaker males does not, as they insistently mated only in HH, despite female status (Table 1). These observations challenge the idea that males perform both tactics or “choose” between them depending on female rejection signaling or on presence and size of opponent males, for example, as it may happen in other loliginid squids. Therefore, it seems that, at least in this population of *D. pleii*, legit sneaker and consort males do not shift willingly between divergent strategies depending on their current mating context.

In contrast, intermediate males of *D. pleii*, unlike typical sneaker and consort males, seem able to interchange between mating tactics depending on the context. Although they typically mated in HH (ca. 91% of times), they were able to switch to MP when the female started laying her eggs in two trials (Table 1 and Supplementary Table S3). Such behavioral changes were observed in different scenarios, and they did not depend on size of the rival male. On trial 37, the intermediate male (ML 157 mm) was placed with a sneaker male (ML 110 mm) and a female (ML 146 mm). Before spawning, the intermediate male mated only in HH, but, as the female started laying egg strings on the substrate, it switched between tactics, pairing with the female and mating in MP (Table 1 and Supplementary Table S3). Contrastingly, on trial 33, the intermediate male (ML 135 mm) cohabited the tank with a female (ML 145 mm) and a consort male (ML 193 mm). Before spawning, it mated in HH. Yet, even in the presence of a larger male, the intermediate male still switched to MP when the female started laying the eggs (Table 1 and Supplementary Table S3). Therefore, it looks like mating context can influence the behavior of intermediate males, but it is apparently related to female status instead of size of rival males.

Interestingly, males of *H. bleekeri* may opt for HH when the female is far from spawning (from ca. 50 to 1 h before egg-laying), changing to MP when the female is about to lay eggs (from 15 min until egg-laying) (Iwata et al., 2005). Although sample size was small in that study (i.e., only three out of the six observed males performed such a behavior; Iwata et al., 2005), these results indicate flexible mating behaviors in that species. Male dimorphism was later demonstrated for *H. bleekeri*, with a switch-point of ca. 220–230 mm in ML (Iwata and Sakurai, 2007; Iwata et al., 2015). From the three males displaying the aforementioned flexible mating strategy, just one had an intermediate size (ca. 220 mm), the other two being much larger than the switch-point (ca. 270 and 300 mm). Also, ARTs are suspected to be permanent in *H. bleekeri* (Iwata and Sakurai, 2007; Iwata et al., 2015). Therefore, the behavior observed in *H. bleekeri* should correspond to a distinct strategy, not related to a transition between male morphs, as observed in *D. pleii*.

Female status (as defined above) seems like a suitable candidate to explain the adoption of different tactics by intermediate males of *D. pleii*, as male fertilization success in loliginid squids is thought to be highly associated with sperm deposition site, due to temporal (i.e., timing between mating and fertilization) and spatial differences between both locations (i.e., near the oviduct vs. near the seminal receptacle) (e.g., Apostólico and Marian, 2017, 2018a). Due to the oocyte pathway during the egg-laying process, consort males are believed to be responsible for higher offspring paternity rates than do sneaker males, as sperm attached near the oviduct opening during MP is expected to contact the unfertilized eggs before those attached near the female’s buccal membrane by HH (Supplementary Figure S1G) (e.g., Buresch et al., 2009; Shashar and Hanlon, 2013). Thus, if an intermediate male is able to visually detect that the female is about to or is already laying eggs, it may opt for mating in MP and depositing spermatophores near the female’s oviduct opening, where the sperm will be promptly used for fertilization and has a higher chance of fertilizing the eggs, even though it has to compete with possible rival consort males. Yet, if the female is not ready to lay eggs, it may be preferable to mate in HH and place the spermatophores close to the seminal receptacle, where the released sperm could be maintained viable for an extended time (e.g., Hanlon, 1996; Hanlon et al., 2002).

If such a flexible strategy is advantageous to intermediate males of *D. pleii*, why do sneaker and consort males not interchange between tactics, too? Two hypotheses can be proposed, based on limitations of either male size or ejaculate type. First, body size of sneaker males may be too small for both MP mating – in which the male must hold the female body and insert spermatophores inside her mantle cavity – and for fighting consort males. Maybe after reaching a certain body mass, males are able to both perform MP and at least try competing with consort males. So, body size constraints could hamper consort behaviors by sneaker males. However, this hypothesis alone does not explain why consort males do not attempt HH copulations when the female is far from spawning.

Another plausible explanation resides in ejaculate morphology and functioning. Dimorphic ejaculates show several adaptations to each sperm deposition site, possibly associated with differences in the interval between mating and fertilization, presence of a sperm storage organ, and egg availability between the sites (Apostólico and Marian, 2017, 2018a,b). For example, sneaker and consort ejaculates show differences in morphology (short and club-like vs. elongate and hook-like spermatangia), sperm release mode (slow vs. fast), and sperm swimming behavior (aggregation vs. diffusion), respectively, that are presumably related to the distinct fertilization environments provided by the buccal membrane and mantle cavity. Therefore, theoretically, both types of ejaculates would be functionally suboptimal if sneaker and consort males interchanged between tactics. For example, the consort spermatangium would be too elongate for the buccal membrane, releasing sperm far from the seminal receptacle, and sperm release would be too fast, both characteristics possibly hindering sperm storage in the seminal receptacle (Apostólico and Marian, 2017).

If dimorphic ejaculates are specifically adapted to each deposition site, then how to explain the flexibility in mating behavior reported herein for intermediate males? One explanation could be that the reported flexibility in behavior is just the result of a physiological transition from sneaker to consort phenotype – then, spermatangia transferred during this time window could be suboptimal depending on their type and site of attachment. Suboptimal attachment could explain, for example, why no club-like spermatangia were found in the oviduct membranes of the female in trial 37, after MP mating with an intermediate male (Supplementary Table S3). It is possible that the club-like spermatangium cannot firmly attach itself to the oviduct membranes. Due to their small size, sneaker-like spermatangia could presumptively have less anchorage and attachment potential than consort-like ones, given their smaller ejaculatory apparatus and cement body (e.g., Marian, 2012a, b; Marian et al., 2012). If this is the case, then they could be more easily flushed from the female’s mantle cavity. In trials 29 and 33, an inadequate attachment could also have happened due to intermediate spermatophores implanted near the seminal receptacle of the female (Supplementary Table S3). Intermediate spermatangia are disproportionally larger than the typical club-like ones found in that particular site. Further investigations accessing paternity rates of these intermediate males could help us understand if suboptimal attachment of spermatangia in unconventional sites may hamper the fertilization success of intermediate males.

Another explanation for the flexibility in mating behavior in intermediate males involves the fact that these males have a transition from sneaker- to consort-like ejaculates in their reproductive system (Apostólico and Marian, 2018b). Within this spermatangia gradient, the intermediate spermatangium type has consort-like morphology – i.e., elongate and hook-like (Supplementary Figure S1I) – but sneaker-like sperm – i.e., with self-swarming (Apostólico and Marian, 2018b). This indicates that the transition in spermatangium morphology happens earlier than in sperm swimming behavior (Apostólico and Marian, 2018b). Thus, when attempting HH copulations long before spawning, intermediate males may still have sneaker sperm in their spermatangia. In turn, when attempting MP mating, although sperm would still be sneaker-like, the hook-like spermatangium (from everted intermediate spermatophores) would be functionally suited for the mantle cavity site. Therefore, intermediate males could benefit from interchanging tactics during the transition from sneaker to consort phenotype. Present data on spermatangia type in each female site seem to corroborate this hypothesis, as intermediate males can transfer intermediate spermatophores to both female sites (Supplementary Table S3). Also, although most of the intermediate males investigated herein transferred only sneaker-like spermatophores during mating (Supplementary Table S3), the hypothesis is not invalidated, as these males are likely “early” intermediate males that still had sneaker-like spermatophores in their storage organ (see below). With time, they would start transferring intermediate ejaculates to both female sites when mating in HH and MP. However, to further address this hypothesis, more data on whether intermediate spermatophores are interchangeably placed in both female sites is required. Moreover, additional investigations accessing not only the type of spermatangia and sperm transferred to females during each type of mating, but also analyzing long-term behavior of intermediate males are necessary.

When Apostólico and Marian (2018b) first discovered a few (and rare – but see discussion below) males with sneaker-, intermediate-, and consort-like spermatophores simultaneously in their storage organ (Supplementary Figure S1H), the authors promptly treated them as probable abnormalities. Only after further analyses, e.g., when the authors realized that these spermatophores were spatially separated inside the organ, they proposed that these rare males were in fact an “intermediate” stage in the ontogenetic transition from sneaker to consort phenotypes. According to their spatial distribution in the organ, males must first use all of their sneaker-like spermatophores (the oldest ones, located more anteriorly in the organ), the intermediate ones, and only then start transferring consort-like ejaculates (the newest ones, located more posteriorly in the organ). As aforementioned, most intermediate males investigated in the present study transferred only sneaker-like structures, despite the mating posture adopted. Also, although a few of them transferred intermediate spermatophores, none of them transferred consort-like ejaculates to females (Supplementary Table S3). These findings are congruent with (and provide new evidence on) a transition in ejaculate production proposed by Apostólico and Marian (2018b). Within the ontogenetic hypothesis, “early” intermediate males may have already started their phenotypic transition (thus being able to mate in both HH and MP), but still transfer the remaining sneaker-like ejaculates before transferring intermediate spermatophores. We hypothesize that, with time, intermediate males would eventually use all of their intermediate spermatophores, cease HH, and perform only MP matings, as legit consorts.

At last, it is important to highlight that ca. 18% (12 out of 66) of all males sampled herein were classified as intermediate males after spermatophore and spermatangia analyses, possibly indicating that intermediate males of *D. pleii* may not be as rare as previously believed (5 out of a total sample of 287 males in Apostólico and Marian, 2018b). The former study argued that intermediate males should correspond to a very rapid transitional stage during ontogeny of dimorphic males, thus explaining their rarity. Here, behavioral results have shown that intermediate males typically behave as sneaker males, i.e., they continuously pursue mating with (new or the same) females. So, these observations might be additional evidence that, if intermediate males do not stop mating and transferring spermatophores, evidence of intermediate ejaculates could be underestimated.

An alternative explanation for the numerical difference in intermediate males sampling could be related to the time of year in which each study was conducted. While samples were obtained mostly from late-spring to early-summer in the former study (Apostólico and Marian, 2018b), the present one was carried out from mid- to late-summer (i.e., until the end of the reproductive peak). Within the ontogenetic hypothesis proposed by Apostólico and Marian (2018b), some males benefit from maturing at smaller size and age and acting as sneaker males along the beginning of their reproductive phase, later changing to a consort morph as they reach a certain body size. If the behavioral and morphological transition is triggered along the ongoing reproductive peak of the population, one could expect finding not only an increasing number of intermediate males toward the end of the reproductive peak, but also a lot of “early” intermediate males. This last hypothesis seems plausible in face of present data, as not only more intermediate males were sampled during mid- and late-summer, but most of them still transferred sneaker-like spermatophores to females.

## Concluding Remarks

Although sneakers and consorts of *D. pleii* do not interchange between tactics, intermediate males, in turn, may show a context-dependent tactic expression, as they can play consort tactics during the female egg-laying period. Therefore, when the female is not ready to lay eggs, they may adopt sneaking tactics and place their sperm in a more secure location, i.e., in the female’s seminal receptacle, when it must survive for longer until the female is ready to lay her eggs. However, performing MP could still be more advantageous than HH during spawning, even if the male does not have a complete consort-like ejaculate, given that the male would gain more fertilizations due to the proximity to the site of egg release. Moreover, considering that the intermediate males which performed MP during the trials had previously transferred spermatangia to the buccal membrane of the same female, this combined strategy of playing both mating tactics may guarantee additional fertilizations for intermediate males.

## Data Availability

The datasets generated for this study are available on request to the corresponding author.

## Ethics Statement

Ethical review and approval was not required for the animal study because in Brazil, ethics approval is still not required for experimentation with cephalopods by the CONCEA, but this study has been carried out in accordance with international procedures for the welfare of cephalopods recommended in the literature.

## Author Contributions

JM and LA designed the study, analyzed the data, and wrote and reviewed the manuscript. LA collected the animals, performed the experiments, and obtained the data on morphology.

## Conflict of Interest Statement

The authors declare that the research was conducted in the absence of any commercial or financial relationships that could be construed as a potential conflict of interest. The handling Editor declared a shared committee membership, though no other collaboration, with one of the authors, JM, in the Cephalopod International Advisory Council.

## Supplementary Material

FIGURE S1 Mating system of the loliginid squid *Doryteuthis pleii*. **(A)** Sneaker males (mantle length, ML < 169 mm) adopt head-to-head (HH) mating posture, whereas consort males (ML > 169 mm) adopt male-parallel (MP) mating posture to mate with females. **(B)** Frontal view of the female mouth region, showing the location of the seminal receptacle (blue star). **(C)** Sagittal section of the female’s seminal receptacle, showing club-like spermatangia from sneaker males, attached during HH mating. **(D)** Consort males express stereotyped body patterns (e.g., the exhibition of red stripes along the body, known as “lateral flames”) in agonistic contests with rival males for female monopolization. **(E,F)** During MP mating, consort males attach hook-like spermatangia to the oviduct membranes of females (green star), inside the mantle cavity. **(G)** During egg capsule formation, consort sperm contacts the eggs first, near the oviduct opening (green star), whereas sneaker sperm contacts the eggs when the egg mass travels near the mouth region of the female (blue star), before being deposited on the substrate. **(H)** Spermatophores from intermediate males, showing a possible transition from the sneaker-like (top) to the consort-like morphology (bottom). While sneaker and consort males have one type of spermatophore (the one on the top and on the bottom, respectively), intermediate males may store both types and intermediate spermatophores (middle) altogether. **(I)** Spermatangia from intermediate males. Club-like spermatangium (typical of sneaker males, left), intermediate spermatangium (center), and hook-like spermatangium (typical of consort males, right). All figures originally published in Apostólico and Marian (2018b) and reproduced here with permission.

TABLE S1 Trials performed using one female with either (i) one consort (trials 01–04, *n* = 4) or (ii) one sneaker male (trials 05–10, *n* = 6) of *Doryteuthis pleii* in captivity.

TABLE S2 Trials performed using one female with either (i) one consort and one sneaker male (trials 11–12, *n* = 2), (ii) two sneaker males (trial 13, *n* = 1), or (iii) two consort males (trials 14–18, *n* = 5) of *Doryteuthis pleii* in captivity.

TABLE S3 Trials performed using one female with either (i) one intermediate male (trials 19–25, *n* = 7), (ii) one intermediate and one consort male (trials 26–33, *n* = 8), or (iii) one intermediate and one sneaker male (trials 34–37, *n* = 4) of *Doryteuthis pleii* in captivity.
