# Supplementary figures and images for: Behavior of “Intermediate” Males of the Dimorphic Squid Doryteuthis pleii Supports an Ontogenetic Expression of Alternative Phenotypes
Source: Front Physiol. 2019 Sep 13;10:1180. doi: 10.3389/fphys.2019.01180 (PMC6753871; doi:10.3389/fphys.2019.01180)

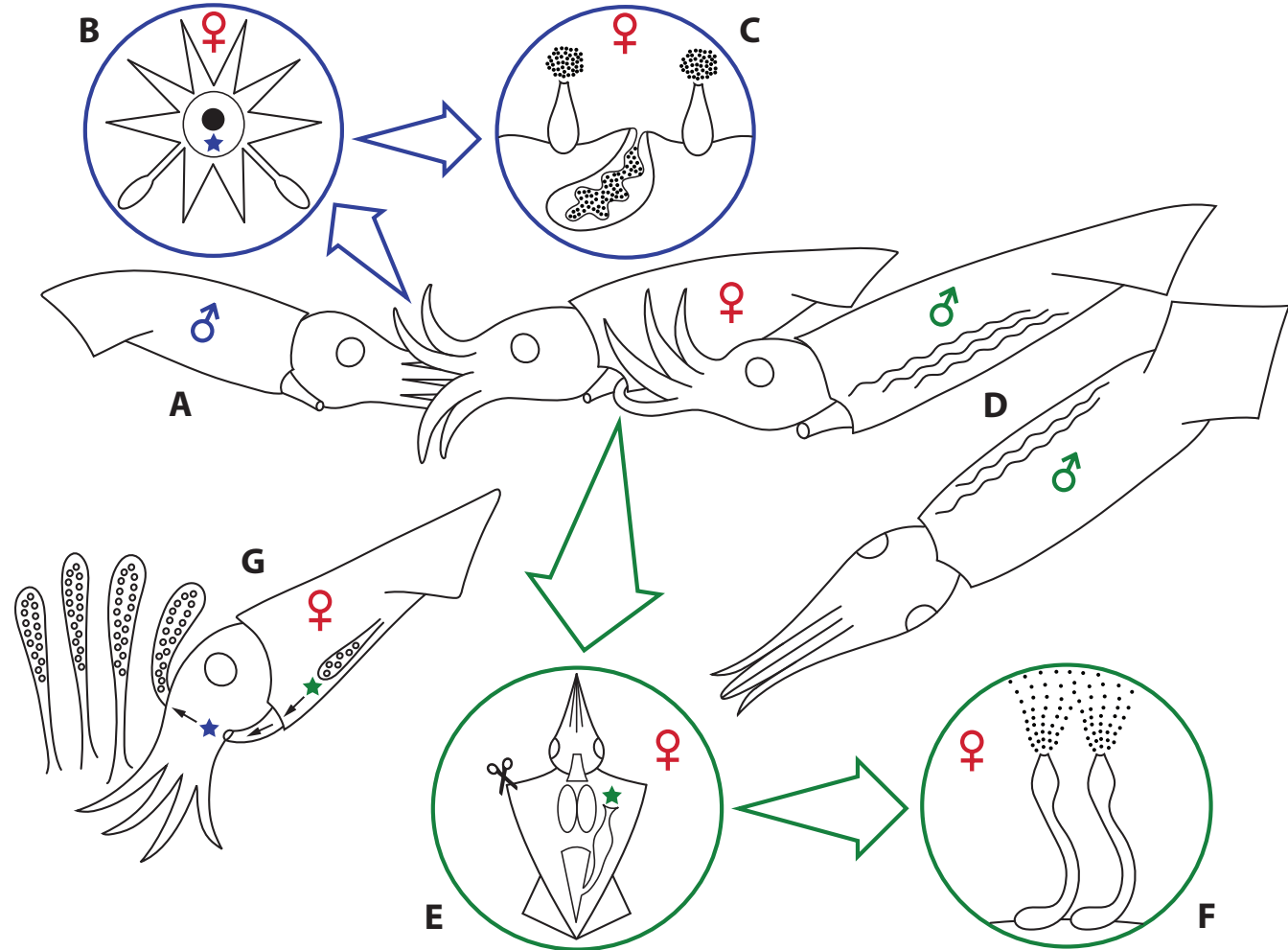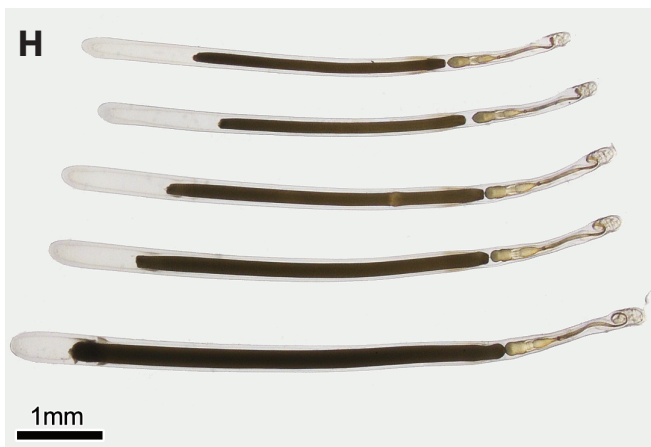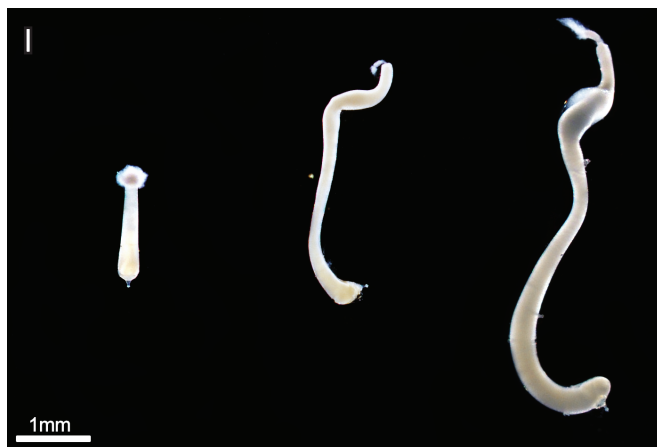

Supplement: FIGURE S1 — Mating system of the loliginid squid Doryteuthis pleii. (A) Sneaker males (mantle length, ML < 169 mm) adopt head-to-head (HH) mating posture, whereas consort males (ML > 169 mm) adopt male-parallel (MP) mating posture to mate with females. (B) Frontal view of the female mouth region, showing the location of the seminal receptacle (blue star). (C) Sagittal section of the female’s seminal receptacle, showing club-like spermatangia from sneaker males, attached during HH mating. (D) Consort males express stereotyped body patterns (e.g., the exhibition of red stripes along the body, known as “lateral flames”) in agonistic contests with rival males for female monopolization. (E,F) During MP mating, consort males attach hook-like spermatangia to the oviduct membranes of females (green star), inside the mantle cavity. (G) During egg capsule formation, consort sperm contacts the eggs first, near the oviduct opening (green star), whereas sneaker sperm contacts the eggs when the egg mass travels near the mouth region of the female (blue star), before being deposited on the substrate. (H) Spermatophores from intermediate males, showing a possible transition from the sneaker-like (top) to the consort-like morphology (bottom). While sneaker and consort males have one type of spermatophore (the one on the top and on the bottom, respectively), intermediate males may store both types and intermediate spermatophores (middle) altogether. (I) Spermatangia from intermediate males. Club-like spermatangium (typical of sneaker males, left), intermediate spermatangium (center), and hook-like spermatangium (typical of consort males, right). All figures originally published in Apostólico and Marian (2018b) and reproduced here with permission. [file Data_Sheet_1.PDF]
