# Supplementary material for: Behavior of “Intermediate” Males of the Dimorphic Squid Doryteuthis pleii Supports an Ontogenetic Expression of Alternative Phenotypes
Source: Front Physiol. 2019 Sep 13;10:1180. doi: 10.3389/fphys.2019.01180 (PMC6753871; doi:10.3389/fphys.2019.01180)
Supplement: TABLE S1 — Trials performed using one female with either (i) one consort (trials 01–04, n = 4) or (ii) one sneaker male (trials 05–10, n = 6) of Doryteuthis pleii in captivity. [file Table_1.DOCX]

**SM Table 1.** Trials performed using one female with either (i) one consort (trials 01-04, n=4) or (ii) one sneaker male (trials 05-10, n=6) of *Doryteuthis pleii* in captivity. Total mantle length (ML) of males and females is expressed in millimeters (mm). For each experiment, the following criteria were analyzed: (1) Spawning: if the female laid eggs after mating (Yes or No); (2) Mating: number of successful mating in each mating posture (HH = head-to-head, MP = male-parallel); (3) Mating-spawning interval: timing (in hours) between the last mating and the spawning event; (4) Spermatophore: type of spermatophore present inside the male’s storage organ (CO = consort spermatophore, SN = sneaker spermatophore). Abbreviations: ♀ = female, ♂CO = consort male, ♂SN = sneaker male, “-”= not applicable, “?” = unknown.

|  | Trial | ML | | Spawning | Mating | Mating-spawning interval | Spermatophore |
| --- | --- | --- | --- | --- | --- | --- | --- |
| ♀+♂CO |  | ♀ | ♂CO |  |  |  |  |
|  |  |  |  |  |  |  |  |
|  | 01 | 217.0 | 255.0 | Yes | 1 MP | < 4h | CO |
|  | 02 | 174.0 | 285.0 | Yes | 1 MP | < 4h | CO |
|  | 03 | 184.0 | 274.0 | Yes | 1 MP | < 4h | CO |
|  | 04 | 134.0 | 178.0 | Yes | 1 MP | < 3h | CO |
|  |  |  |  |  |  |  |  |
| ♀+♂SN |  | ♀ | ♂SN |  |  |  |  |
|  |  |  |  |  |  |  |  |
|  | 05 | 153.0 | 110.0 | No | 1 HH | - | SN |
|  | 06 | 166.0 | 110.0 | No | 1 HH | - | SN |
|  | 07 | 147.0 | 110.0 | No | 1 HH | - | SN |
|  | 08 | 151.0 | 113.0 | Yes | 1 HH | Between 03h to16h | SN |
|  | 09 | 145.0 | 102.0 | Yes | 1 HH | Between 09h to23h | SN |
|  | 10 | 140.0 | 110.0 | Yes | 1 HH | 26h | SN |

**SM Table 2.** Trials performed using one female with either (i) one consort and one sneaker male (trials 11-12, n=2), (ii) two sneaker males (trial 13, n=1), or (iii) two consort males (trials 14-18, n=5) of *Doryteuthis pleii* in captivity. Total mantle length (ML) of males and females is expressed in millimeters (mm). For each experiment, the following criteria were analyzed: (1) Spawning: if the female laid eggs after mating (Yes or No); (2) Agonistic behavior: if the male showed any aggressive display towards the other male in the tank (Yes or No); (3) Mating: number of successful mating in each mating posture (HH = head-to-head, MP = male-parallel); (4) Mating-spawning interval: timing (in hours) between the last mating and the spawning event; (5) Spermatophore: type of spermatophore present inside the male’s storage organ (CO = consort spermatophore, SN = sneaker spermatophore). Abbreviations: ♀ = female, ♂CO = consort male, ♂SN = sneaker male, “-”= not applicable, “?” = unknown.

|  | Trial | ML | | | Spawning | Agonistic behavior | | Mating | | Mating-spawning interval | | Spermatophore | |
| --- | --- | --- | --- | --- | --- | --- | --- | --- | --- | --- | --- | --- | --- |
| ♀+♂SN+♂CO |  | ♀ | ♂SN | ♂CO |  | ♂SN | ♂CO | ♂SN | ♂CO | ♂SN | ♂CO | ♂SN | ♂CO |
|  |  |  |  |  |  |  |  |  |  |  |  |  |  |
|  | 11 | 141.0 | 152.0 | 258.0 | No | No | No | 1 HH | ? | - | - | SN | CO |
|  | 12 | 170.0 | 113.0 | 243.0 | No | No | No | 1 HH | ? | - | - | SN | CO |
|  |  |  |  |  |  |  |  |  |  |  |  |  |  |
| ♀+♂SN1+♂SN2 |  | ♀ | ♂SN1 | ♂SN2 |  | ♂SN1 | ♂SN2 | ♂SN1 | ♂SN2 | ♂SN1 | ♂SN2 | ♂SN1 | ♂SN2 |
|  |  |  |  |  |  |  |  |  |  |  |  |  |  |
|  | 13 | 160.0 | 102.0 | 137.0 | Yes | No | No | 2 HH | 3 HH | 24h | 0h (during spawning) | SN | SN |
|  |  |  |  |  |  |  |  |  |  |  |  |  |  |
| ♀+♂CO1+♂CO2 |  | ♀ | ♂CO1 | ♂CO2 |  | ♂CO1 | ♂CO2 | ♂CO1 | ♂CO2 | ♂CO1 | ♂CO2 | ♂CO1 | ♂CO2 |
|  |  |  |  |  |  |  |  |  |  |  |  |  |  |
|  | 14 | 157.0 | 258.0 | 235.0 | No | Yes | Yes | ? | ? | - | - | CO | CO |
|  | 15 | 171.0 | 243.0 | 221.0 | No | Yes | Yes | ? | ? | - | - | CO | CO |
|  | 16 | 185.0 | 206.0 | 270.0 | No | Yes | Yes | ? | ? | - | - | CO | CO |
|  | 17 | 166.0 | 237.0 | 223.0 | No | Yes | Yes | ? | ? | - | - | CO | CO |
|  | 18 | 174.0 | 275.0 | 281.0 | No | Yes | Yes | ? | ? | - | - | CO | CO |

**SM Table 3.** Trials performed using one female with either (i) one intermediate male (trials 19-25, n=7), (ii) one intermediate and one consort male (trials 26-33, n=8), or (iii) one intermediate and one sneaker male (trials 34-37, n=4) of *Doryteuthis pleii* in captivity. Total mantle length (ML) of males and females is expressed in millimeters (mm). For each experiment, the following criteria were analyzed: (1) Spawning: if the female laid eggs after mating (Yes or No); (2) Agonistic behavior (only when there were 2 males in the tank): if the male showed any aggressive display towards the other male in the tank (Yes or No); (3) Mating: number of successful mating in each mating posture (HH = head-to-head, MP = male-parallel); (4) Mating-spawning interval: timing (in hours) between the last mating and the spawning event; (5) Spermatophore: type(s) of spermatophore present inside the male’s storage organ (CO = consort spermatophore, INT = intermediate spermatophore, SN = sneaker spermatophore); (6) Spermatangium: type(s) of spermatangium implanted in the oviduct membranes (OV) and/or near the seminal receptacle (SR) (CO = consort spermatangia, INT = intermediate spermatangia, SN = sneaker spermatangia). The oviduct membranes and seminal receptacle regions were only inspected in females that laid eggs. As explained in the text, intermediate males were first assorted as either ‘sneaker’ or ‘consort’ males during the trials, based on their body size. Their identity as intermediate males was only confirmed after inspection of their spermatophores and spermatangia morphology. Abbreviations: ♀ = female, ♂CO = consort male, ♂INT = intermediate male, ♂SN = sneaker male, “-”= not applicable, “?” = unknown, “None” = no spermatangia observed in the oviduct membranes, although male mated in MP.

|  | Trial | ML | | | Spawning | Agonistic behavior | | Mating | | Mating-spawning interval | | Spermatophore | | Spermatangium | |
| --- | --- | --- | --- | --- | --- | --- | --- | --- | --- | --- | --- | --- | --- | --- | --- |
|  |  |  |  |  |  |  |  |  |  |  |  |  |  | OV | SR |
| ♀+♂INT |  | ♀ | ♂INT |  |  |  |  | ♂INT | | ♂INT | | ♂INT | |  |  |
|  |  |  |  |  |  |  |  |  | |  | |  | |  |  |
|  | 19 | 159.0 | 178.0 |  | No | - | | 1 HH | | - | | SN,INT,CO | | ? | ? |
|  | 20 | 120.0 | 156.0 |  | No | - | | 1 HH | | - | | SN,INT | | ? | ? |
|  | 21 | 147.0 | 138.0 |  | No | - | | 1 HH | | - | | SN,INT | | ? | ? |
|  | 22 | 170.0 | 138.0 |  | No | - | | 1 HH | | - | | SN,INT | | ? | ? |
|  | 23 | 154.0 | 138.0 |  | No | - | | 1 HH | | - | | SN,INT | | ? | ? |
|  | 24 | 130.0 | 153.0 |  | No | - | | 1 HH | | - | | INT,CO | | ? | ? |
|  | 25 | 155.0 | 132.0 |  | Yes | - | | 2 HH | | Between 03 to 16h | | SN,INT | | - | SN |
|  |  |  |  |  |  |  | |  | |  |  |  |  |  |  |
| ♀+♂INT+♂CO |  | ♀ | ♂INT | ♂CO |  | ♂INT | ♂CO | ♂INT | ♂CO | ♂INT | ♂CO | ♂INT | ♂CO |  |  |
|  |  |  |  |  |  |  |  |  |  |  |  |  |  |  |  |
|  | 26 | 141.0 | 132.0 | 235.0 | No | No | Yes | 1 HH | ? | - | - | SN,INT | CO | ? | ? |
|  | 27 | 156.0 | 144.0 | 206.0 | No | Yes | Yes | ? | ? | - | - | INT,CO | CO | ? | ? |
|  | 28 | 184.0 | 162.0 | 237.0 | No | Yes | Yes | ? | ? | - | - | INT,CO | CO | ? | ? |
|  | 29 | 160.0 | 149.0 | 214.0 | Yes | Yes | Yes | 1 HH | 2 MP | < 3h | 0h (during spawning) | INT,CO | CO | CO | INT |
|  | 30 | 160.0 | 138.0 | 206.0 | Yes | No | No | 1 HH | 1 MP | 2h | < 2h | SN,INT | CO | CO | SN |
|  | 31 | 137.0 | 138.0 | 217.0 | Yes | No | No | 1 HH | 1 MP | 48h | < 3h | SN,INT | CO | CO | SN |
|  | 32 | 132.0 | 138.0 | 191.0 | Yes | No | No | 1 HH | ? | 19h | ? | SN,INT | CO | CO | SN |
|  | 33 | 145.0 | 135.0 | 193.0 | Yes | No | No | 3 HH | 1 MP | 00h30h | 0h (during spawning) | INT, CO | CO | INT, CO | INT |
|  |  |  |  |  |  |  |  | 1 MP |  | 0h (during spawning) |  |  |  |  |  |
|  |  |  |  |  |  |  |  |  |  |  |  |  |  |  |  |
| ♀+♂INT+♂SN |  | ♀ | ♂INT | ♂SN |  | ♂INT | ♂SN | ♂INT | ♂SN | ♂INT | ♂SN | ♂INT | ♂SN |  |  |
|  |  |  |  |  |  |  |  |  |  |  |  |  |  |  |  |
|  | 34 | 143.0 | 157.0 | 110.0 | Yes | No | No | 2 HH | 1 HH | 32h | 34h | SN,INT,CO | SN | - | SN |
|  | 35 | 147.0 | 138.0 | 155.0 | Yes | No | No | 1 HH | ? | Between 05 to 17h | ? | SN,INT | SN | - | SN |
|  | 36 | 153.0 | 138.0 | 152.0 | Yes | No | No | 1 HH | 2 HH | 10h | 28h | SN,INT | SN | - | SN |
|  | 37 | 146.0 | 157.0 | 110.0 | Yes | No | No | 1 HH | 2 HH | 4h | 7h | SN,INT,CO | SN | None | SN |
|  |  |  |  |  |  |  |  | 1 MP |  | 0h (during spawning) |  |  |  |  |  |
|  |  |  |  |  |  |  |  |  |  |  |  |  |  |  |  |
